# Chemical Regimes and PMF-Resolved Sources Linked to Acute PM_10_ Ecotoxicity in Luanda, Angola

**DOI:** 10.3390/toxics14090773

**Published:** 2026-08-30

**Authors:** Alan Victor da Silva, Estela D. Vicente, Ana M. Sánchez de la Campa, Yago Cipoli, Diogo N. Cardoso, Susana Loureiro, Anabela Leitão, Manuel Feliciano, Célia Alves

**Affiliations:** 1 Department of Environment and Planning, Centre for Environmental and Marine Studies (CESAM), University of Aveiro, 3810-193 Aveiro, Portugal; 2 Department of Earth Sciences, Associate Unit CSIC-University of Huelva “Atmospheric Pollution”, Centre for Research in Sustainable Chemistry (CIQSO), University of Huelva, E21071 Huelva, Spain; 3 Department of Biology, Centre for Environmental and Marine Studies (CESAM), University of Aveiro, 3810-193 Aveiro, Portugal; 4 Separation, Chemical Reaction, and Environmental Engineering Laboratory (LESRA), Agostinho Neto University, Av. Ho Chi Minh nº 201, Luanda, Angola; 5 CIMO, LA SusTEC, Instituto Politécnico de Bragança, Campus Santa Apolónia, 5300-253 Bragança, Portugal

**Keywords:** compositional clustering, trace metals, urban aerosol, Microtox, *Aliivibrio fischeri*, African megacity

## Abstract

In rapidly expanding African cities, the ecotoxicological relevance of PM_10_ remains largely unresolved, especially where particle mass, chemical composition and emission sources are rarely assessed together. Here, 116 daily PM_10_ samples collected in Luanda between 27 June and 5 November 2023 were tested using the Microtox^®^ *Aliivibrio fischeri* assay on aqueous extracts. Chemical speciation was combined with centred log-ratio (CLR) compositional clustering to identify recurring chemical regimes, while Positive Matrix Factorisation (PMF) was used to resolve the corresponding emission sources. Toxicity was moderate but highly variable: Toxic units after 15 min (TU_15_) ranged from 0.84 to 8.54, with changes in toxicity units between 5 and 15 min (ΔTU) varying from −1.51 to +4.84. Only 3 of the 116 samples showed TU_15_ < 1, and none reached TU_15_ ≥ 10. The strongest responses occurred under a metal-enriched chemical regime (C2), characterised by elevated Zn, Pb and Cd concentrations, which showed the highest mean TU_15_ (4.22) and ΔTU (1.52). This regime coincided with PMF evidence identifying non-ferrous metallurgy plus galvanised/metal scrap handling and burning as the factor most strongly associated with toxicity (ρTU_15_ = 0.63; ρΔTU = 0.78), followed by a Ni-rich metallurgical factor with possible aviation influence (ρTU_15_ = 0.51). Conversely, a marine/ionic chemical regime (C3) showed lower, stable toxicity, consistent with negative marine aerosol associations. These findings show that integrating chemical regimes with PMF-resolved sources identifies metal-related emissions as the main drivers of PM_10_ ecotoxicity.

## 1. Introduction

Particulate matter with an aerodynamic diameter ≤ 10 µm (PM_10_) remains a major public health concern, with the 2021 World Health Organisation (WHO) recommending a 24 h guideline value of 45 µg m^−3^ [1]. PM_10_ concentrations remain high in many rapidly expanding cities of the Global South [2,3]. Even in cities with declining trends, such as Beijing, levels still exceed the WHO guidance [4]. Against this backdrop, the mean PM_10_ concentration observed in Luanda in 2023 (59.3 µg/m^3^) confirms particle pollution as a relevant concern in African urban settings [5]. Short-term PM_10_ exposure has been associated with increased daily mortality across 652 cities worldwide [6]. The State of Global Air 2025 report further estimated that air pollution contributed to 7.9 million deaths worldwide in 2023, largely driven by particulate pollution [7]. Yet monitoring remains sparse in the most affected regions, with lower-middle- and low-income countries contributing only about 6% of global air quality measurements despite representing almost half of the world’s population [8,9].

PM is a chemically heterogeneous mixture whose biological and ecotoxic effects depend on mass, physicochemical properties and emission sources [10,11,12]. In particular, redox-active metals and combustion-related organic fractions, including polycyclic aromatic hydrocarbons (PAHs) and quinones, have been linked to reactive oxygen species generation, oxidative stress and inflammatory responses, while organic carbon and PAHs have also been associated with ecotoxic and cytotoxic responses [11,13]. Thus, chemical characterisation and source apportionment are essential for identifying harmful components and targeting mitigation [10,14,15]. Chemical characterisation alone cannot fully capture the biological potency of real-world particle mixtures, which may depend on bioavailability, sample preparation and interactions among constituents [16,17]. Effect-based approaches therefore complement chemical and source-based analyses by capturing responses to bioavailable and combined mixture properties that are not apparent from mass concentration alone [16,18]. This is particularly relevant because atmospheric particles and particle-bound contaminants can deposit into terrestrial and aquatic environments, affecting biota [19,20].

The Microtox^®^ luminescence inhibition assay using *Aliivibrio fischeri* is useful for PM screening because it is rapid and reproducible, provides a quantifiable endpoint, requires small sample amounts, and is responsive to PAH and metal-rich fractions associated with PM toxicity [17,21]. However, the response of *A. fischeri* represents a specific acute bacterial endpoint and therefore captures only one dimension of PM toxicity. Complementary bioassays addressing different biological endpoints and test systems can provide a broader assessment of particle toxicity [16,18]. Previous applications of *A. fischeri*-based assays to PM in São Paulo (Brazil) and Bragança (Portugal) have linked ecotoxicity to biomass-burning contributions and toxic PM episodes [14,15]. Together, these considerations support the integration of ecotoxicological screening with chemical speciation and source apportionment, especially in African urban settings where high PM burdens coexist with limited monitoring [7,9].

Recent monitoring in central Luanda revealed frequent exceedances of WHO guidelines for PM_10_, PM_2.5_ and NO_2_ [5]. Analyses of the same campaign showed that PM_10_ is dominated by traffic-related emissions, with contributions from dust, marine aerosol, industrial and metallurgical activities, and secondary aerosol [22]. An elemental assessment revealed strong anthropogenic enrichment of PM_10_-bound toxic metal(loid)s, particularly Sb and Cd, accompanied by a very high potential ecological risk (RI = 4360 ± 2440) and substantial inhalation risks, with both the non-carcinogenic hazard index (HI > 2) and carcinogenic risk (CR > 10^−3^) exceeding accepted thresholds [23]. Despite recent progress in characterising pollutant levels, chemical composition and source contributions, it remains unclear how these signatures translate into biological effects in Luanda. The present work addresses this gap by linking the acute ecotoxicity of aqueous PM_10_ extracts with chemically speciated PM_10_ and PMF-resolved sources in Luanda.

## 2. Materials and Methods

### 2.1. Study Area, Sampling and Chemical Characterisation

**Figure 1:**
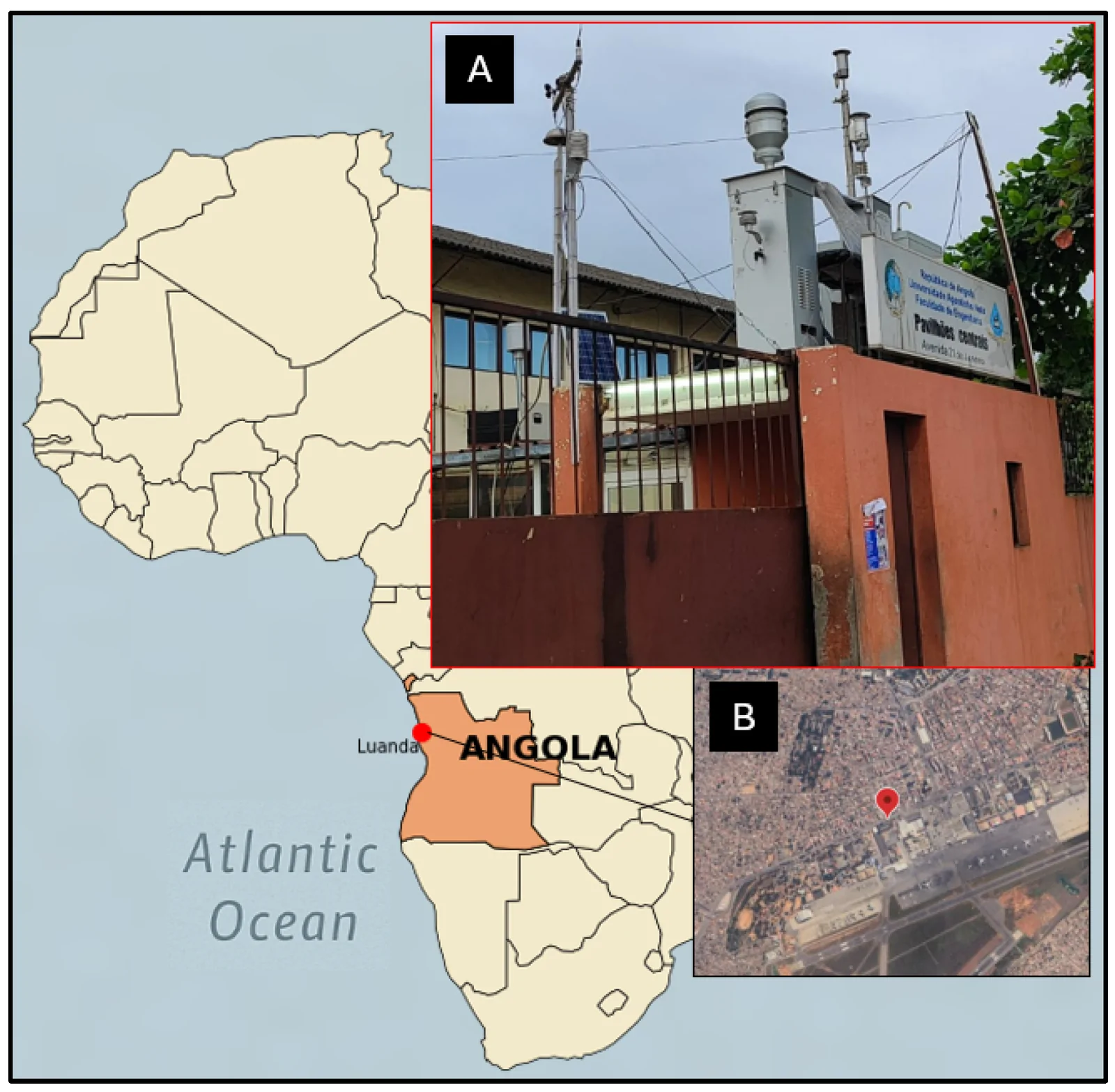
Location of the sampling site in Luanda, Angola (−8°50′49.77″ S, 13°13′51.11″ E). (**A**) Equipment at the Faculty of Engineering, Agostinho Neto University; (**B**) satellite view of the sampling site and surrounding area.

The sampling site was located in a densely urbanised area, close to 4 de Fevereiro International Airport and characterised by substantial nearby road traffic (Figure 1). This study analysed 116 daily PM_10_ samples collected in central Luanda, Faculty of Engineering, Agostinho Neto University, between 27 June and 5 November 2023. Daily PM_10_ samples were collected by gravimetric methods using high-volume quartz filters (AirFlow PM_10_-HVS, AMS Analitica^®^, Pesaro, Italy; Pall^®^, New York, NY, USA) and low-volume Teflon filters (Echo PM, Tecora^®^, Cogliati, Italy; Pall^®^). Filters were conditioned, weighed under controlled conditions, and stored at low temperature until analysis. Full operational details and co-located instrumentation are provided in the companion publications [5].

PM_10_ samples were chemically characterised for (i) water-soluble inorganic ions by ion chromatography, (ii) anhydrosugars/polyols by high-performance anion-exchange chromatography with pulsed amperometric detection (HPAEC-PAD), (iii) carbonaceous fractions (OC/EC) using a thermal–optical protocol inspired by EUSAAR-II, and (iv) major/minor elements using ICP-OES/ICP-MS. Daily source apportionment was performed using the U.S. EPA Positive Matrix Factorisation model (PMF v5.0.14), based on paired concentration and uncertainty matrices prepared in accordance with standard guidance for observations below the method detection limit and for missing values [22]. The measured chemical species were subsequently grouped and converted, where appropriate, to reconstruct the main PM_10_ mass components.

### 2.2. PM_10_ Mass Reconstruction

The PM_10_ mass balance was reconstructed by combining the measured carbonaceous, inorganic ionic, and elemental fractions, together with an estimate of particle-bound water. Because the analytical techniques quantify different chemical fractions and, in some cases, overlapping forms of the same element, the reconstruction procedure was designed to avoid double counting.

OC and EC were determined by thermal–optical analysis in transmittance mode. Organic matter (OM) was estimated from the measured OC concentration using an OC-to-OM conversion factor of 1.6, consistent with values recommended for urban aerosols [24,25], whereas EC was included directly as measured.

Elemental concentrations determined by ICP-OES and ICP-MS were converted stoichiometrically to oxide-equivalent concentrations using the corresponding molecular-to-element mass ratios. The oxide forms used for this calculation were selected as representative abundant/stable oxide forms for mass-reconstruction purposes, using stoichiometric conversion factors reported in the literature [26]. These oxide equivalents were used solely for mass reconstruction and should not be interpreted as direct identification of the molecular forms present in PM_10_. For example, Ca may occur in different chemical forms, including carbonates, sulfates and nitrates [27].

Water-soluble inorganic ions were determined independently by ion chromatography. The measured ionic species included SO_4_^2−^, Cl^−^, NO_2_^−^, PO_4_^3−^, NO_3_^−^, F^−^, Br^−^, Li^+^, Na^+^, K^+^, Mg^2+^, Ca^2+^, and NH_4_^+^. To prevent double counting, whenever an element quantified by ICP-OES/ICP-MS was already represented in the reconstructed mass through its corresponding oxide equivalent, its water-soluble ionic fraction determined by ion chromatography was not added separately to the reconstructed PM_10_ mass. Thus, only SO_4_^2−^, Cl^−^, NO_2_^−^, NO_3_^−^, F^−^, Br^−^, and NH_4_^+^ were added. Nevertheless, these water-soluble fractions were retained as analytically measured species and are reported separately where relevant.

Anhydrosugars and polyols determined by HPAEC-PAD were not included separately in the mass balance because they are organic constituents and therefore already accounted for in the OM fraction. Particle-bound water was not determined analytically but was estimated using a Köhler-based approach, from SO_4_^2−^, Cl^−^, NO_3_^−^, NH_4_^+^, Na^+^ and K^+^ concentrations and RH-dependent hygroscopic growth factors (Table S1) [28,29,30]. Water uptake for each species was calculated as follows:

*W_i_* = *C_i_* [*HGF_i_*(*RH*) − 1] (1)

where *W_i_* is the water associated with species *i*, *C_i_* is its measured concentration, and *HGF_i_*(*RH*) is the hygroscopic growth factor at the corresponding relative humidity. Individual contributions were summed. Accordingly, this fraction is hereafter referred to as estimated particle-bound water.

All the species included and the calculation approach are presented in Table S2. The reconstructed PM_10_ mass was therefore calculated as follows:

PM_10,reconst._ = OM + EC + element-derived oxide equiv. + non-overlapping WSI + estimated particle-bound water. (2)

The unaccounted mass was calculated as the difference between the gravimetrically determined PM_10_ mass and the sum of the reconstructed components.

### 2.3. Ecotoxicity Assay

Two 14 mm diameter punches from each quartz filter were immersed in 4 mL of ultrapure water (Millipore Corporation, Billerica, MA, USA), sonicated twice for 15 min, and filtered through a 0.2 µm PVDF filter with polypropylene housing, yielding the mother extract, defined as 100%. The ecotoxicity of the aqueous PM_10_ extracts was evaluated using the Microtox^®^ bioluminescence inhibition assay with *Aliivibrio fischeri*, and a Microtox Model 500 analyser (Modern Water Inc., New Castle, DE, USA). The mother extracts were tested using a 1:2 serial dilution scheme (nine two-fold dilutions spanning approximately 0.32–81.9% *v*/*v*) prepared in 2% NaCl, which also served as the control/blank solution. Measurements were conducted at 15 °C, with bioluminescence readings after 5 and 15 min of exposure [31,32]. EC_50_ (the extract concentration causing 50% inhibition of bacterial bioluminescence) values were calculated from the concentration–gamma regression following the Microtox data-reduction procedure described in Supplementary Methods S1. Results were expressed as toxicity units, calculated as

TU = 100/EC_50_. (3)

and as

ΔTU = TU_15_ − TU_5_ (4)

Operationally, TU < 1 was treated as negligible-to-slight acute toxicity, whereas TU ≥ 10 indicated high acute toxicity and TU > 100 very high acute toxicity [33].

### 2.4. Quality Control and Data Curation

Each EC_50_ was derived from the log-linear concentration–gamma regression using only concentration points that met the Microtox validity criteria (positive, dose-consistent gamma response); points flagged as invalid by the data-reduction procedure were excluded from the fit. Assays were retained for quantitative analysis only when supported by sufficient valid concentration points and a coherent dose–response. Samples whose extracts did not reach 50% inhibition at the highest tested concentration but yielded an otherwise valid curve were reported as TU < 1 (EC_50_ > maximum tested), whereas assays with too few valid points or documented technical anomalies (control/blank drift, instrument-communication errors, or handling contamination) were excluded. Doubtful assays were re-run where sample material allowed (33 samples), and the retained replicate was used. After curation, 116 daily samples yielded valid EC_50_ estimates at both exposure times. In the final dataset, EC_50_ values were supported by 4–7 valid concentration points (only three samples at a three-point minimum), indicating the robustness of the reported toxicity units.

### 2.5. Data Processing

Data preprocessing and visualisation were performed in Python (3.13.15) using pandas (2.2.2), NumPy (2.0.2), Matplotlib (3.10.0), SciPy (1.16.3) and scikit-learn (1.6.1). To emphasise day-to-day changes in relative chemical composition rather than absolute concentration, a compositional-profile (COMP) approach was adopted, consistent with compositional data analysis principles [34] and previous applications to pollutant profiles and air pollution datasets [35,36].

Negative artefacts and values below the species-specific limit of detection (LOD) were treated as missing values. Chemical species were retained only when at least 80% of daily observations across the campaign were above the LOD. For the retained species, remaining missing values were replaced by the species-wise median concentration, preserving the daily sample structure while reducing the influence of episodic peaks. For each sampling day *i* and chemical species *j*, the imputed concentration *x_ij_* was converted into a within-day composition “*P_ij_*” by closure across the selected species:

(5) $$P_{\mathrm{ij}}=\frac{x_{\mathrm{ij}}}{\sum_{k=1}^{n}x_{\mathrm{kj}}}$$

To enable log-ratio transformation, a small constant pseudocount ε (10^−6^) was then added uniformly to all components to avoid zeros. The resulting compositions were transformed using the centred log-ratio (CLR):

(6) $${\mathrm{CLR}}_{\mathrm{ij}}=\ln\left(P_{\mathrm{ij}}+\varepsilon \right)-\frac{1}{n}\sum_{k=1}^{n}\ln\left(P_{\mathrm{ij}}+\varepsilon \right)$$

which expresses each species as a log-ratio relative to the day-specific geometric mean and accounts for the closure constraint [34]. CLR-transformed variables were subsequently robustly scaled (median-centred and scaled by the interquartile range) prior to clustering analyses.

Sample-level clustering was performed on principal component (PC) scores derived from the robust-scaled CLR matrix. PCs were retained until at least 85% of total variance was explained, resulting in 13 PCs that accounted for approximately 86% of the variance. PCA was implemented in scikit-learn using svd_solver = ‘full’, retaining components that preserved the dominant compositional structure while avoiding low-variance components that reduced clustering stability in sensitivity checks. Hierarchical clustering was then applied to the PCA scores using Ward linkage and Euclidean distance Figure S1. To reduce subjectivity in selecting the number of clusters, solutions from *k* = 2 to 10 were evaluated using the Silhouette coefficient, Calinski–Harabasz index and Davies–Bouldin index (Table S3) [37,38,39]. Based on these internal validation criteria, solutions with four to six clusters were identified as the main candidate range. For each candidate solution (*k* = 4–6), 500 random subsamples containing approximately 80% of the observations were generated without replacement [40]. The complete clustering procedure was repeated for each subsample, and agreement between the resulting partition and the corresponding full-dataset solution was quantified using the adjusted Rand index (ARI) (Table S4) [41]. The five-cluster solution showed the highest resampling stability and was therefore retained. For this solution, cluster-specific stability was further assessed using Jaccard similarity [42]. In each resampling iteration, each cluster from the full-data solution was matched to the resampled cluster yielding the highest Jaccard similarity [42]. The retained five-cluster solution corresponded to a dendrogram cut at d = 20 (criterion = ‘distance’; Figure S2), yielding clusters C1–C5. Temporal coherence and interpretative consistency were considered additional, rather than primary, supporting criteria. Cluster profiles were subsequently characterised in CLR space using cluster–global contrasts:

(7) $${\Delta CLR}_{c,j}={\bar{CLR}}_{c,j}-{\bar{CLR}}_{\mathrm{global},j}$$

where *c* denotes the cluster index. Each cluster was therefore interpreted as a recurring relative compositional signature, defined by species over- or under-represented relative to the campaign-wide PM_10_ composition rather than by absolute concentration. Positive values indicate over-representation and negative values under-representation. The top species in each direction were reported. Associations between chemical species and ecotoxicity (TU) were screened using Spearman’s rank correlation (ρ_s_) [43].

## 3. Results and Discussion

**Figure 2:**
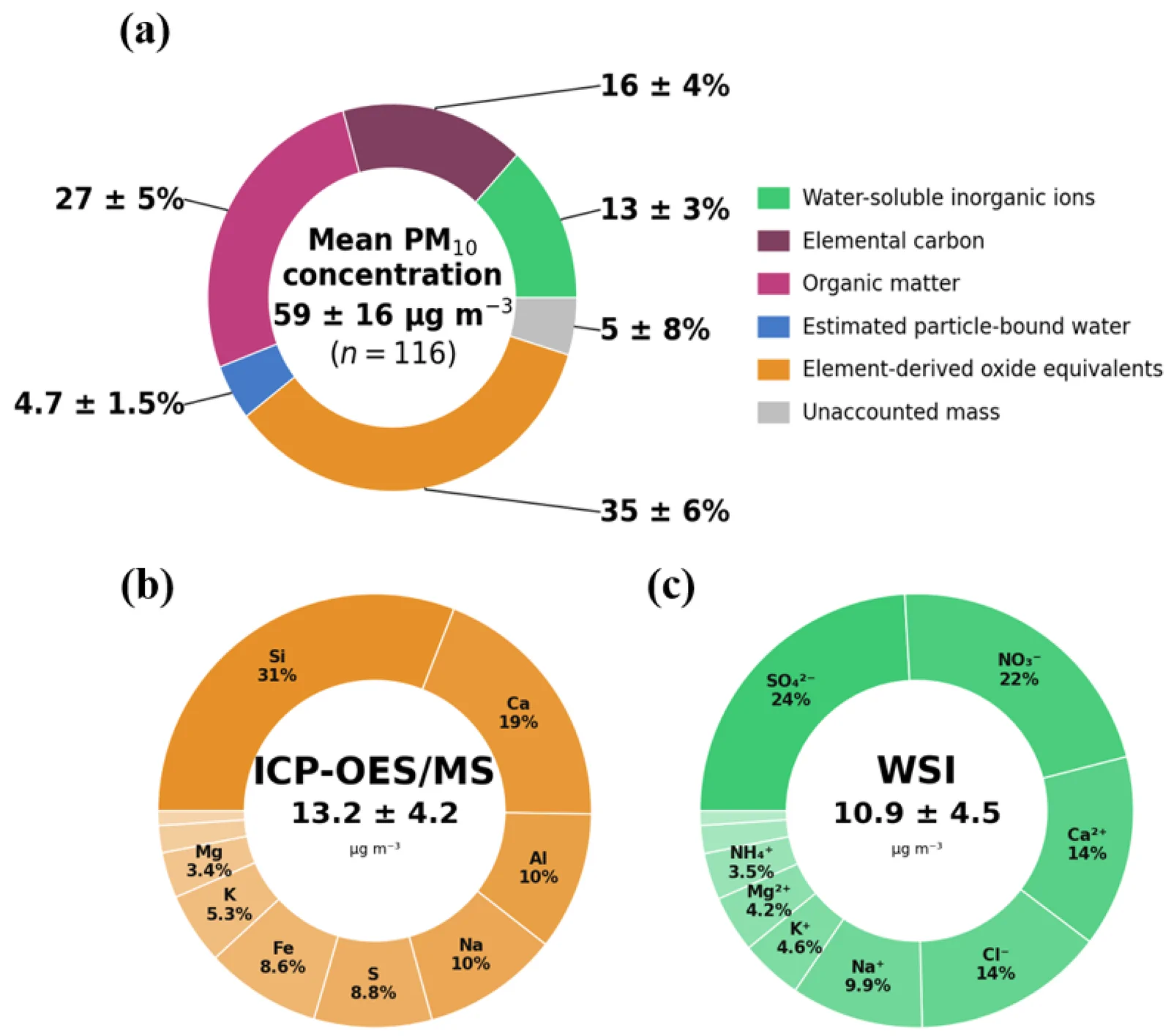
Campaign-average PM_10_ mass reconstruction and composition of the inorganic analytical fractions in Luanda. (**a**) Relative contributions of organic matter (OM), elemental carbon (EC), element-derived oxide equivalents, non-overlapping water-soluble inorganic ions (WSI), estimated particle-bound water, and unaccounted mass to gravimetrically determined PM_10_. (**b**) Relative elemental composition determined by ICP-OES/MS and (**c**) relative composition of WSI determined by ion chromatography. Elemental concentrations used in the mass reconstruction were converted stoichiometrically to representative oxide equivalents; these quantities do not imply direct identification of molecular or mineralogical phases. Water-soluble ions were measured independently by ion chromatography, and ionic fractions overlapping with elements already represented in the ICP-derived oxide-equivalent fraction were not added separately to the reconstructed mass. Panels (**b**,**c**) show the complete analytical composition of each technique, irrespective of this overlap treatment. Values at the centre of panels indicate the mean ± SD mass concentration of the corresponding analytical fraction (µg m^−3^), while segment labels indicate the relative contribution of each species within that fraction. Particle-bound water was estimated using the Köhler-based approach. Species contributing <3% were grouped as “Other elements” in panel (**b**) and “Other ions” in panel (**c**). Colours are used only for visual distinction among components and do not represent an additional variable or quantitative scale.

The campaign-average PM_10_ mass reconstruction is shown in Figure 2. Carbonaceous material represented a major fraction of the reconstructed aerosol mass, with organic matter (OM) and elemental carbon (EC) accounting for 27% ± 5% and 16% ± 4% of PM_10_, respectively. This places Luanda towards the upper range commonly reported for urban PM_10_ carbonaceous fractions [5,44,45,46]. Element-derived oxide equivalents accounted for 35% ± 6% of PM_10_, with SiO_2_, CaO, Al_2_O_3_ and NaO as the dominant components. Non-overlapping WSI represented 13% ± 3% of the PM_10_, with SO_4_^2−^, NO_3_^−^ and Cl^−^ accounting for 11% collectively. Estimated particle-bound water accounted for 4.7 ± 1.5% of PM_10_. It was calculated using a Köhler-theory-based approach rather than determined directly. Anhydrosugars and polyols represent 2.6% of OM, with levoglucosan representing 29% of this specific fraction. The remaining unaccounted mass (5.3% ± 8.4%) may reflect unquantified carbonates, residual particle-bound water not captured by the estimation, unmeasured organic or secondary aerosol components, and volatilisation losses [47].

The weekly and monthly mean variations in the concentrations of the major chemical classes of PM_10_ are depicted in Figure S3a,b. During the cool, largely rain-free “cacimbo” season (June–September), the Angolan coast is influenced by the Benguela current and persistent low stratus cloud and fog [48]. These seasonal conditions may favour mineral dust mobilisation and resuspension [49], although this meteorological data was not directly evaluated in the present study. Consistent with these conditions, element-derived oxide equivalents exhibited higher contributions for PM_10_ in June–July (≈35.5%) and lower values from August to October (≈33.0%). September, the most episodic month of the campaign, also precedes the onset of the hot, wet season (October–May) associated with the southward migration of the Intertropical Convergence Zone (ITCZ), and Angola can experience a late summer rainfall maximum [49,50]. As convection and precipitation strengthen, wet deposition (scavenging) becomes an effective atmospheric sink for aerosols [51], while WSI decreased from 20% in June–July to ~17% in August–October before increasing again in November (20%). Carbonaceous fractions were comparatively stable over the campaign; however, OC/EC (1.33 ± 0.47) became more variable in September [22]. The unaccounted mass fraction increased from August (~7%) to September (~10%), then decreased in October (~5%) and remained moderate in November (~7%). No clear weekly pattern was observed, although Sunday showed a distinct compositional profile compared with the other weekdays [5,52].

**Figure 3:**
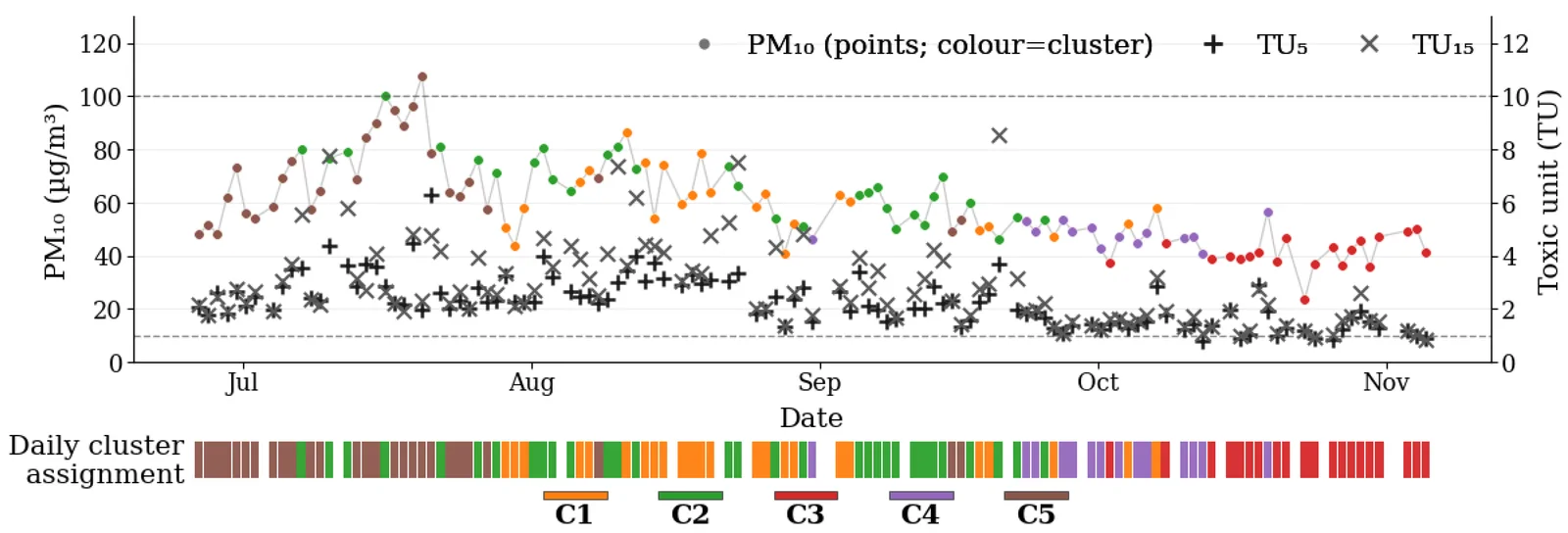
PM_10_ clusters and toxicity timeline: PM_10_ (points; colour = cluster, C1–C5) with TU_5_ (+) and TU_15_ (X) overlaid on a secondary axis; dashed lines mark TU thresholds (1 and 10). The lower barcode indicates cluster membership by day.

The seasonal structure became clearer after clustering daily PM_10_ composition (Figure 3; Table 1). Internal validation identified *k* = 4–6 as the main competing range, with the Silhouette coefficient favouring *k* = 5, the Calinski–Harabasz index *k* = 4 and the Davies–Bouldin index *k* = 6 (Table S4). Among these competing solutions, *k* = 5 showed the greatest resampling stability (mean ARI = 0.599; Table S4) and was therefore retained. Cluster-specific stability was heterogeneous, with mean Jaccard similarities decreasing in the order C3 (0.87) > C5 (0.79) > C4 (0.68) > C1 (0.59) > C2 (0.50) (Table S5). Late June and July were more frequently characterised by C5, showing relative enrichment in NH_4_^+^, K, and levoglucosan, consistent with the period of greatest wildfire activity recorded nationwide [22]. From August to late September, the composition alternated mainly between C2 (relative enrichment in Zn, Pb, Cd, and Ni) and C1 (relative enrichment in Ba, NH_4_^+^, EC, Sb, and Cu), indicating stronger anthropogenic and metal-related influence. Although C4 showed intermediate resampling stability (mean Jaccard similarity = 0.68; Table S5), its compositional fingerprint was less pronounced, with comparatively small positive ΔCLR values for enriched species (Table 1). C4 occurred mainly between the earlier C1/C2- and later C3-dominated periods (Figure 3), overlapping temporally with the statistically significant shift in PM_10_ previously reported for September during the same campaign [5]. Together with the onset of Ni, Pb and levoglucosan depletion, these features suggest a transitional compositional regime [22]. From mid-October to November, the increased prevalence of C3 (Na, Cl^−^, SO_4_^2−^ and Mg enriched) suggests a relatively stronger marine influence than during the earlier C1/C2-dominated period.

Toxicity units ranged from TU_5_ = 0.80–6.28 to TU_15_ = 0.84–8.54, with ΔTU (TU_15_ − TU_5_) from −1.51 to +4.84, showing pronounced temporal variability in both magnitude and exposure-time response. Overall, Luanda falls predominantly within the 1–10 TU band, with only 3 of the 116 samples showing TU_15_ < 1 and none reaching TU_15_ ≥ 10. Cross-study comparisons should be treated as indicative because they combine different particle-size fractions and endpoints (e.g., TU_15_ vs. TU_30_) and, in some cases, different extraction matrices. In São Paulo, aqueous PM_2.5_ extracts screened using a kinetic *Aliivibrio fischeri* assay were all toxic, with TU_30_ = 1.7–7.1 (mean 3.7; [15]), placing Luanda within a similar order of magnitude despite endpoint differences. In Istanbul, PM_2.5–10_ extracts analysed with Microtox^®^ Model 500 showed an episodic toxicity threshold of TU_15_ = 8.73 [53], close to Luanda’s maximum TU_15_ of 8.54. By contrast, PM_10_ aqueous extracts from Bragança, Portugal, ranged from TU = 0.3 to 27.5, including very toxic samples [14], indicating that stronger acute responses can occur under different source and atmospheric settings.

Late June showed moderate and closely aligned toxicity values (TU_15_ 1.76–2.77; ΔTU −0.15 to +0.12), indicating little exposure-time amplification and coinciding with the C5 regime (NH_4_^+^, K and levoglucosan enriched). July displayed the greatest intra-month dispersion (TU_15_ 1.90–7.77), including a marked increase on July 10th (TU_15_ 7.77; ΔTU +3.42). From August to late September, when daily composition alternated mainly between C2 (Zn, Pb, Cd, Ni enriched) and C1 (Ba, NH_4_^+^, EC, Sb, Cu enriched), TU remained highly variable and ΔTU was predominantly positive, with the campaign maximum on September 20th (TU_15_ 8.54; ΔTU +4.84). In October and early November, when samples were more frequently classified as C3 (Na, Cl^−^, SO_4_^2−^ and Mg enriched), toxicity levels were lower and more stable (TU_15_ 0.84–3.18; ΔTU −0.04 to +0.65). Overall, TU_15_ exceeded TU_5_ for most samples, supporting TU_15_ as the primary endpoint, with ΔTU retained as complementary metrics [32]. Negative ΔTU values occurred in 16 of 116 samples (13.8%), ranging from −1.51 to −0.01 TU (median = −0.09 TU). Most were small in magnitude, with 12 of the 16 negative values (75%) within 0.20 TU of zero. Given their generally small magnitude, values close to zero may partly reflect analytical variability associated with the Microtox^®^ procedure rather than a biologically meaningful difference in toxicity [32].

**Figure 4:**
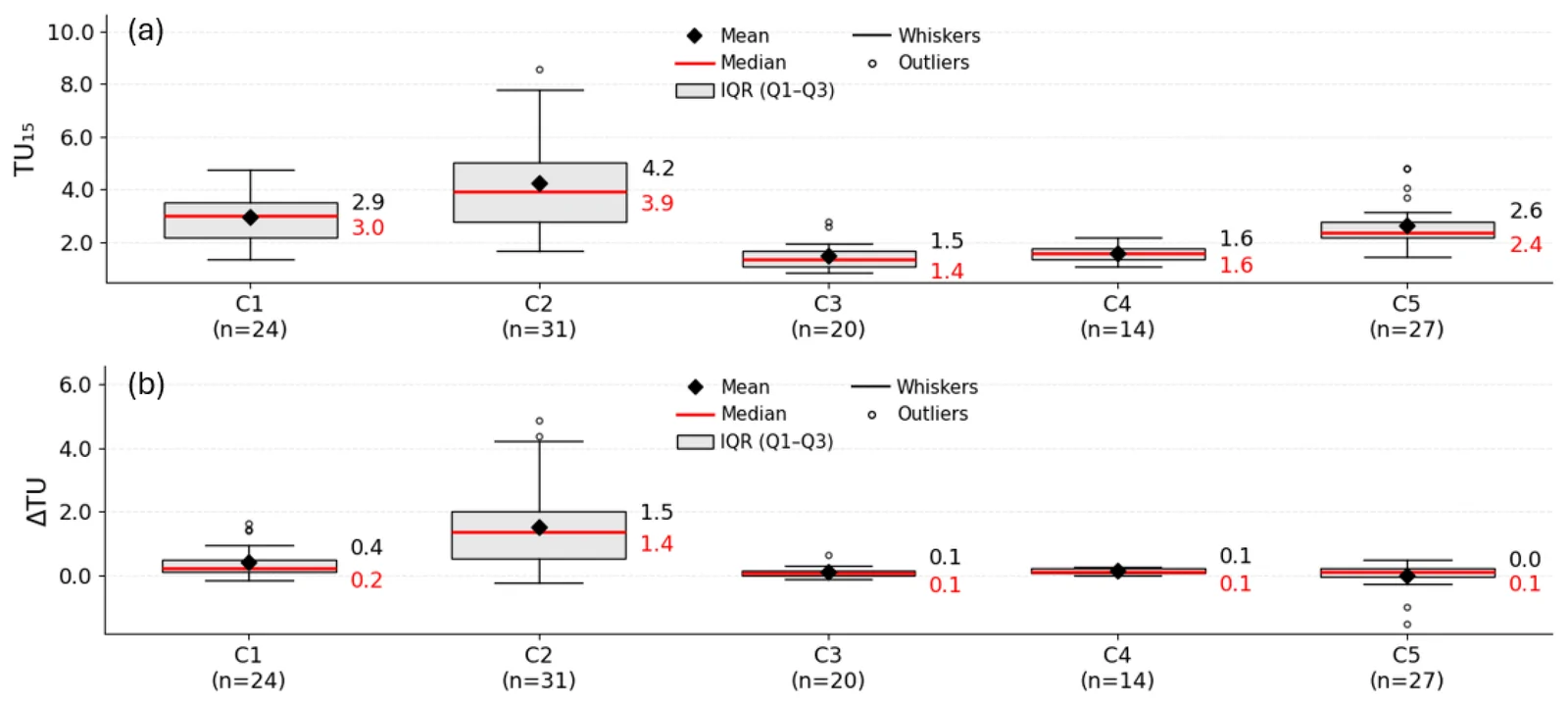
Boxplots by compositional cluster (C1–C5): (**a**) TU_15_; (**b**) ΔTU.

Figure 4 shows that C2 (Zn, Pb, Cd, Ni enriched) was the most pronounced toxicity regime, with the highest TU_15_ (4.22), the widest dispersion and the strongest exposure-time dependence (ΔTU = 1.52). The C2 fingerprint aligns with the strongest positive correlates: Cd ranks among the top TU_15_ correlates (ρ = 0.81), with Pb and Zn also showing strong positive associations (ρ = 0.76 and 0.65, respectively). ΔTU is most strongly correlated with Pb, Zn and Cd (ρ = 0.72–0.76; Figure S4). These results are consistent with a predominantly metal-driven luminescence response in *A. fischeri*, as metals can bind to bacterial cell-surface functional groups and disrupt cellular metabolism, leading to reduced bioluminescence [54]. The positive ΔTU under C2 further suggests time-intensified inhibition, consistent with the behaviour of metallic toxicants [55]. Importantly, mixture effects should not be assumed to be purely additive. Depending on metal pairing, effect level and matrix chemistry, both synergistic and antagonistic responses have been reported in *Aliivibrio*/Microtox-type assays. For example, mixtures of As, Cd, Hg and Pb have been reported to exhibit a summed TU < 1, consistent with synergism [56]. Conversely, Cu(II)–Cd(II)–Pb(II)–Zn(II) mixtures tested across multiple ratios yielded antagonistic behaviour (summed TU > 1) in an immobilised-cell fibre-optic microoptode format [54]. C1 (Ba, NH_4_^+^, EC, Sb, Cu enriched) exhibits intermediate magnitude (TU_15_ = 2.95) with modest exposure-time dependence (ΔTU = 0.42). Its tracers are reflected among moderately significant positive correlates: NH_4_^+^, EC, and Sb are associated with TU_15_ (ρ ≈ 0.56–0.57 for EC and Sb), while Sb, Cu, and EC remain significant ΔTU correlates (ρ ≈ 0.48, 0.47, and 0.37, respectively). The smaller ΔTU in C1 suggests a weaker increase in inhibition between 5 and 15 min compared with C2. One hypothesis that remains to be tested is that the C1 matrix may constrain metal bioaccessibility, for example, through binding to organic components or association with less soluble phases, thereby potentially contributing to the weaker time-dependent response [57]. C5 (NH_4_^+^, K and levoglucosan enriched) also lies in the intermediate TU_15_ (2.63) but shows minimal net exposure-time dependency (ΔTU = 0.03). Within its fingerprint, K and levoglucosan are prominent significant correlates of TU_15_ (ρ ≈ 0.65–0.64), while K shows the largest ΔTU association among the C5 tracers (ρ = 0.21), consistent with a biomass-burning influence. However, these species are best treated as source tracers rather than direct drivers of toxicity. Microtox-type inhibition under biomass-burning conditions may reflect water-extractable combustion-related organics and other co-emitted compounds that co-vary with these tracers [11,13]. However, poorly water-soluble particle-bound organics, including many PAHs, may be incompletely represented by the aqueous extraction procedure. In contrast, C3 (Na, Cl^−^, SO_4_^2−^ and Mg enriched) defines the low-magnitude cluster (TU_15_ = 1.47; ΔTU = 0.11) and aligns with the main significant negatives in the Spearman screens, notably Na for TU_15_ (ρ = −0.22) and Na and Cl^−^ for ΔTU (ρ = −0.36 and −0.26, respectively). The lower toxicity observed in C3 may reflect its distinct chemical composition, although the marine physiology of *A. fischeri* means that higher salinity/ionic strength could influence the assay response and contribute to the lower apparent inhibition [58]. Finally, C4—the least distinct cluster, characterised by depletion in Ni, Pb, and levoglucosan—shows low responses (TU_15_ = 1.59; ΔTU = 0.15).

Daily PM_10_ source apportionment reproduced PM_10_ mass well (R^2^ = 0.95, RMSE = 3.95 µg/m^3^) and resolved six sources [22]. These comprised traffic (exhaust plus non-exhaust, 37.6%), non-ferrous metallurgy plus galvanised/metal scrap handling and burning (10.8%), an Ni-centred metallurgical source with a possible aviation overlay (6.4%), crustal material/resuspended dust (16.5%), marine aerosol (13.1%), and secondary aerosol mixed with biomass burning (SA + BB, 15.5%).

**Figure 5:**
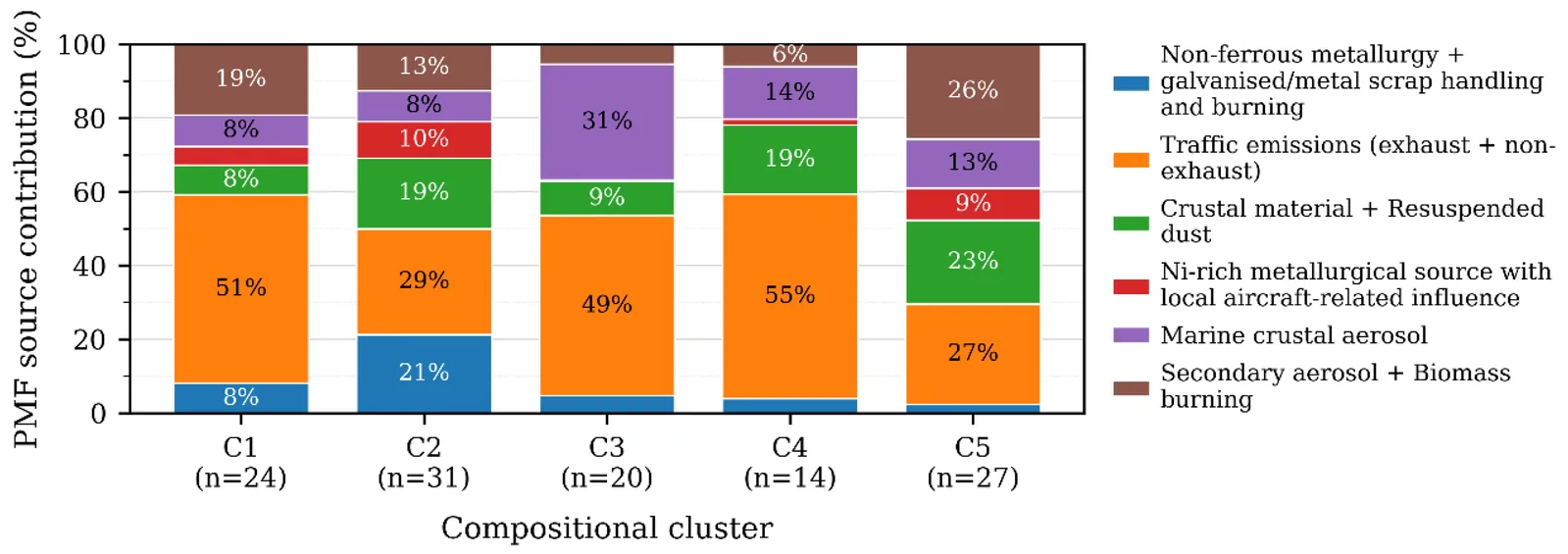
PMF-resolved source contribution profiles across compositional clusters in daily PM_10_ samples from Luanda, Angola.

**Figure 6:**
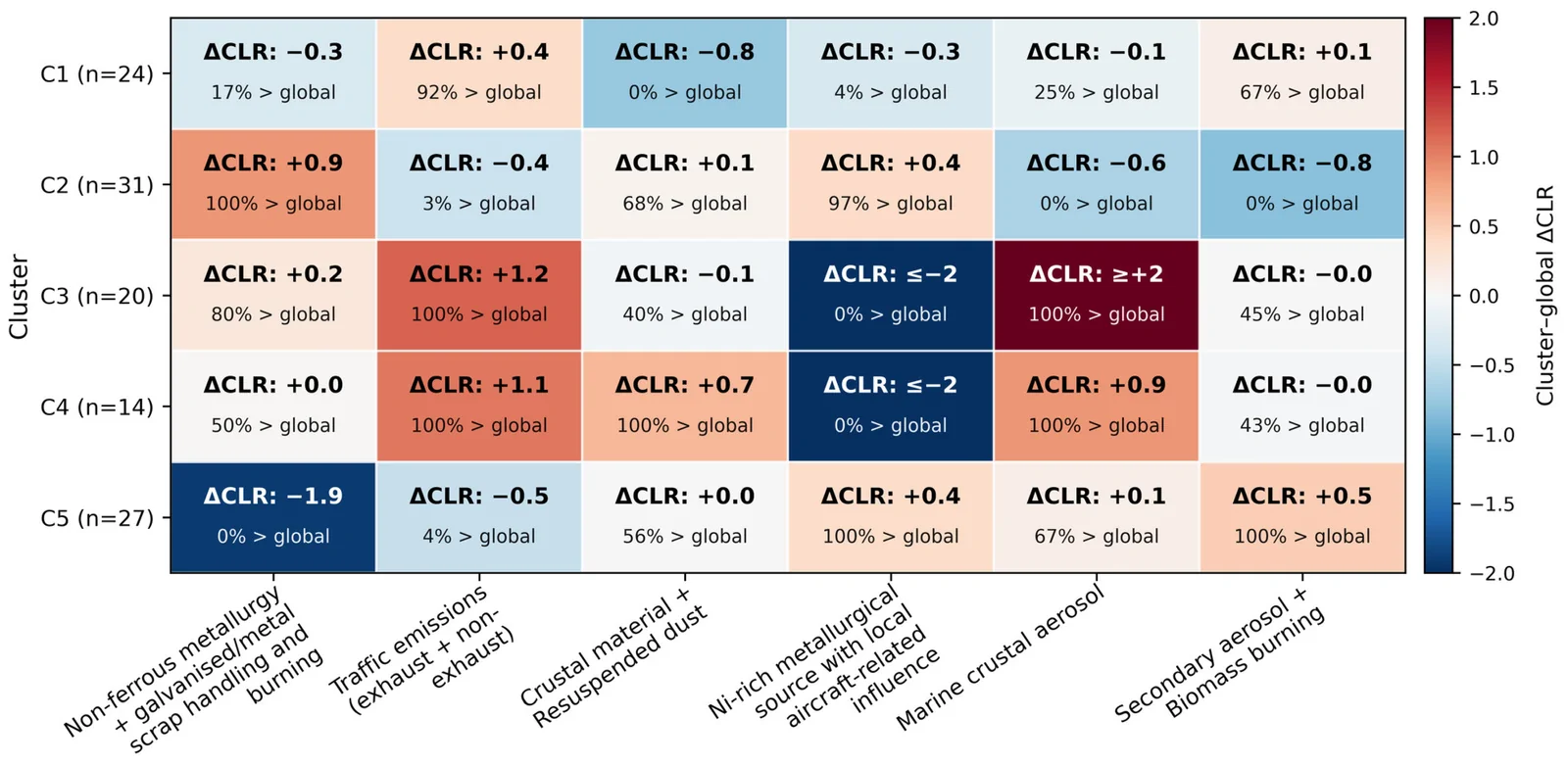
Heatmap of the relative enrichment and depletion of PMF-resolved source contributions across the five chemical-composition clusters. Colours and bold cell annotations represent cluster–campaign ΔCLR values calculated from the daily PMF source contributions, indicating the direction and magnitude of relative source enrichment (positive) or depletion (negative) within each cluster. Percentages indicate the proportion of samples in each cluster for which the CLR-transformed contribution of that PMF source exceeded the campaign-wide reference. Thus, ΔCLR should be interpreted as the primary measure of cluster-level relative enrichment or depletion, whereas the percentages provide complementary information on the within-cluster consistency of the corresponding pattern.

The contribution of these sources across compositional clusters is shown in Figure 5, Figures S5 and S6. SA + BB was most influential from late June to mid-July, consistent with the period of high wildfire activity, and aligned with the C5 regime (enriched in NH_4_^+^, K and levoglucosan) [22]. Its contribution reached ~26% in C5 and declined to near-residual levels from late August onwards. Toxicologically, SA + BB was positively associated with TU_15_ (ρ = 0.47) but showed almost no association with ΔTU (ρ = 0.03), indicating limited exposure-time amplification. The non-ferrous metallurgy plus galvanised/scrap handling and burning factor was more intermittent but clearly expressed under the C2 regime (Zn, Pb and Cd enriched), where its contribution reached 21%, compared with <8% in the other clusters (Figure 5). This factor showed the strongest associations with toxicity (ρTU_15_ = 0.63; ρΔTU = 0.78) and was clearly enriched in C2 in CLR source space (ΔCLR = +1.0; 81% of samples above the campaign-wide reference; Figure 6). This factor was also highly stable in the original PMF analysis, with 100% bootstrap consistency and stable DISP behaviour [22]. C2 was also enriched in the Ni-centred metallurgical factor (ΔCLR = +0.6; 74% of samples above the campaign-wide reference), which showed the second strongest positive association with toxicity (ρTU_15_ = 0.51; ρΔTU = 0.29). Together, these patterns support a robust association between C2 and a broader metal-related source regime, while source co-variation, mixture effects and contributions from unmeasured components preclude attribution of the observed toxicity to any single source or chemical component [16,17]. This factor weakened late in the campaign, consistent with Ni depletion and the shift towards C3–C4, where ΔCLR ≤ −2 and contributions fell below 1%. It was the second strongest positive toxicity correlate (ρTU_15_ = 0.51; ρΔTU = 0.29).

Traffic was the dominant and most persistent PM_10_ source, increasing from 27% in C5 to 55% in C4 and remaining prominent in C1. However, it showed weak, non-significant negative associations with ecotoxicity (ρTU_15_ = −0.12; ρΔTU = −0.06). This weak traffic–toxicity association should be interpreted cautiously because non-exhaust traffic and resuspended dust share overlapping urban mechanical tracers and are difficult to separate in PMF [59,60,61]. In this context, the moderate dust–toxicity association (ρTU_15_ = 0.40; ρΔTU = 0.24) may reflect not purely crustal material, but a mixed urban resuspension signal carrying traffic-related non-exhaust particles. Marine aerosol was negatively associated with both TU_15_ and ΔTU (ρ = −0.24 and −0.30, respectively). It was most clearly enriched in C3, where its contribution reached 31%, with ΔCLR ≥ +2 and 100% of samples above the campaign-wide reference. This pattern agrees with the lower and more stable toxicity observed under the C3 ionic/marine regime.

## 4. Conclusions

This study evaluated the acute ecotoxicity of aqueous PM_10_ extracts from Luanda using the Microtox^®^ *Aliivibrio fischeri* assay and interpreted day-to-day variability through chemically defined regimes and PMF-resolved sources. Toxicity was generally moderate, with TU_15_ mostly within the 1–10 range and no sample reaching TU_15_ ≥ 10, but it showed marked temporal variability. Toxicity was comparatively stable in late June but became more variable and time-amplified during the Cacimbo, particularly from July to September. Toxicity then decreased as the PM_10_ mixture became less metal-enriched and increasingly dominated by marine/ionic conditions.

Composition was central to the ecotoxic response. The Zn-, Pb-, Cd- and Ni-enriched regime showed the highest TU_15_ and strongest ΔTU, supporting a strong association between metal enrichment and the observed inhibition response. At the source level, the non-ferrous metallurgy plus galvanised/metal scrap handling and burning factor showed the strongest association with ecotoxicity, followed by the Ni-rich metallurgical factor with a possible local aircraft-related influence. However, these associations do not establish metals or the identified metallurgical sources as direct drivers of toxicity, as their causal contribution cannot be confirmed without direct information on metal speciation and bioaccessibility. Source co-variation, mixture effects and unmeasured components may also have contributed to the observed response. However, the contribution of metals cannot be confirmed as causal without direct information on their speciation and bioaccessibility. By contrast, the marine/ionic regime coincided with lower and more stable toxicity. Biomass-burning-influenced conditions showed intermediate toxicity but weak delayed response, suggesting that exposure-time amplification was more characteristic of metal-enriched regimes than of tracer-defined combustion signatures.

These findings should be interpreted with caution because the analysis relied on a single bacterial species and a single acute endpoint, aqueous extraction, and correlation-based inference. Accordingly, the ecotoxicological patterns should be regarded as screening-level evidence and cannot be generalised to potential effects on other aquatic or terrestrial organisms without further testing. Future work should combine metal speciation and bioaccessibility measurements with a battery of bioassays covering different trophic levels and biological endpoints, together with targeted approaches to investigate the mechanisms underlying the observed toxicity, while retaining the regime-based framework developed here. This approach could support more targeted air quality management in Luanda by identifying the source- and composition-defined conditions associated with greater ecotoxic hazard. The EC_50_/TU estimates rely on a log-linear concentration–response fit; for a small number of samples near the assay detection limit, EC_50_ approached or exceeded the highest tested concentration, and the corresponding TU should be interpreted as a lower-bound indication of toxicity.

## Figures and Tables

**Table 1:** CLR-based compositional fingerprints of clusters. For each cluster, the table reports the five species with the highest positive ΔCLR values (Enriched) and the five species with the lowest negative ΔCLR values (Depleted).

Cluster	Enriched (ΔCLR)	Depleted (ΔCLR)
C1	Ba (0.427); NH_4_^+^ (0.368); EC (0.348); Sb (0.276); Cu (0.221)	Y (−0.324); Se (−0.215); Li (−0.205); Al (−0.193); Th (−0.189)
C2	Zn (0.836); Pb (0.828); Ni (0.516); Cd (0.469); Bi (0.271)	NH_4_^+^ (−0.504); Na (−0.392); Cl^−^ (−0.366); S (−0.213); U (−0.207)
C3	Na (0.624); Cl^−^ (0.552); Mg (0.398); U (0.359); Se (0.290)	Ni (−1.066); K (−0.783); Levoglucosan (−0.506); Pb (−0.358); Gd (−0.349)
C4	Na (0.216); Fructose (0.200); Ca (0.199); La (0.195); U (0.190)	Ni (−0.821); Zn (−0.676); Levoglucosan (−0.508); Pb (−0.426); NH_4_^+^ (−0.404)
C5	NH_4_^+^ (0.642); K (0.564); Ni (0.497); Levoglucosan (0.305); Y (0.218)	Zn (−0.738); Pb (−0.488); Sn (−0.427); Sb (−0.346); Cu (−0.275)

## Data Availability

The raw data supporting the conclusions of this article will be made available by the authors on request.

## Supplementary Materials

The following supporting information can be downloaded at https://www.mdpi.com/article/10.3390/toxics14090773/s1, Table S1: Relative-humidity-dependent hygroscopic growth factors (HGF) used for the estimation of particle-bound water in PM_10_; Table S2: Species included in the PM_10_ mass reconstruction, calculation approach, and treatment of analytical overlap; Table S3: Quantitative assessment of cluster-number solutions using internal validity indices (k = 2–10); Table S4: Resampling-based assessment of global stability for four-, five-, and six-cluster solutions using 80% of the observations; Table S5: Resampling-based cluster-specific stability of the retained five-cluster solution under 80% subsampling; Supplementary Method S1: Microtox^®^ data reduction; Figure S1: PCA biplots of COMP/CLR data by cluster; Figure S2: PCA–Ward dendrogram of daily PM_10_ chemical composition; Figure S3: Temporal variation in PM_10_ chemical composition by major classes; Figure S4: Spearman rank correlations ($\rho_{s}$) between each chemical species and TU15 (a) and ΔTU (b); Figure S5: Temporal evolution of CLR-relative contributions of PMF-resolved sources in daily PM_10_ samples from Luanda, Angola; Figure S6: Heatmap of CLR-relative PMF source contributions across daily PM_10_ chemical regimes.

## Abbreviations

The following abbreviations are used in this manuscript:

ARI	Adjusted Rand index
BB	Biomass burning
CLR	Centred log-ratio
COMP	Compositional-profile approach
CR	Carcinogenic risk
DISP	Displacement analysis
EC	Elemental carbon
EC_50_	Effective concentration causing 50% inhibition
EPA	United States Environmental Protection Agency
EUSAAR-II	European Supersites for Atmospheric Aerosol Research II
HI	Hazard index
HPAEC-PAD	High-performance anion-exchange chromatography with pulsed amperometric detection
ICP-MS	Inductively coupled plasma mass spectrometry
ICP-OES	Inductively coupled plasma optical emission spectrometry
ITCZ	Intertropical Convergence Zone
LOD	Limit of detection
OC	Organic carbon
OM	Organic matter
PAHs	Polycyclic aromatic hydrocarbons
PC	Principal component
PCA	Principal component analysis
PM	Particulate matter
PM_2.5_	Particulate matter with an equivalent aerodynamic diameter ≤ 2.5 µm
PM_10_	Particulate matter with an equivalent aerodynamic diameter ≤ 10 µm
PMF	Positive Matrix Factorisation
PVDF	Polyvinylidene fluoride
RH	Relative humidity
RI	Potential ecological risk index
RMSE	Root mean square error
SA + BB	Secondary aerosol and biomass burning
SD	Standard deviation
TU	Toxicity unit
TU_5_	Toxicity unit after 5 min of exposure
TU_15_	Toxicity unit after 15 min of exposure
TU_30_	Toxicity unit after 30 min of exposure
ΔTU	Difference between TU_15_ and TU_5_
WHO	World Health Organization
WSI	Water-soluble inorganic ions
