# Supplementary material for: Chemical Regimes and PMF-Resolved Sources Linked to Acute PM10 Ecotoxicity in Luanda, Angola
Source: Toxics. 2026 Aug 30;14(9):773. doi: 10.3390/toxics14090773 (PMC13611758; doi:10.3390/toxics14090773)
Supplement: Supplementary file 1 [file toxics-14-00773-s001.zip › toxics-4485508-supplementary.pdf]

Chemical regimes and PMF-resolved sources linked to acute PM_{10} ecotoxicity in Luanda, Angola

Alan Victor da Silva[1*], Estela Vicente[1], Ana Sánchez de la Campa[2], Yago Cipoli[1], Diogo N. Cardoso[3], Susana Loureiro[3], Anabela Leitão[4], Manuel Feliciano[5], Célia Alves[1*]

[1] CESAM — Centre for Environmental and Marine Studies, Department of Environment and Planning, Campus Universitário de Santiago, University of Aveiro, 3810-193 Aveiro, Portugal;

[2] Associate Unit CSIC-University of Huelva "Atmospheric Pollution", Centre for Research in Sustainable Chemistry - CIQSO, Department of Earth Sciences, Faculty of Experimental Sciences, University of Huelva, 21071 Huelva, Spain;

[3] CESAM - Centre for Environmental and Marine Studies, Department of Biology, Campus Universitário de Santiago, University of Aveiro, 3810-193 Aveiro, Portugal;

[4] LESRA – Separation, Chemical Reaction, and Environmental Engineering Laboratory, Agostinho Neto University, Av. Ho Chi Minh nº 201, Luanda, Angola;

[5] CIMO, LA SusTEC, Instituto Politécnico de Bragança, Campus de Santa Apolónia, 5300-253 Bragança, Portugal;

[*] alanvisi@gmail.com; celia.alves@ua.pt

Supplementary Materials:

This document contains supplementary materials that provide additional information and details on the methodologies, data, and results discussed in the main body of the article.

Table S1. Relative-humidity-dependent hygroscopic growth factors (HGF) used for the estimation of particle-bound water in PM_{10}.

RH (%)	*SO_4^{2-}*	*NO_3^-*	*NH_4^+*	*Cl^-*	*Na^+*	*K^+*
50	1.1	1.1	1.1	1.0	1.0	1.0
60	1.2	1.2	1.2	1.0	1.0	1.0
70	1.3	1.3	1.2	1.1	1.1	1.1
80	1.5	1.3	1.2	1.2	1.2	1.2
90	1.7	1.4	1.3	1.3	1.3	1.3

Note: HGF values were applied according to ambient relative humidity in the Köhler-based estimation of particle-bound water.

Table S2. Species included in the PM_{10} mass reconstruction, calculation approach, and treatment of analytical overlap.

Measured quantity	*Method*	*Representation in mass balance*	*Treatment*
OC	Thermal-optical transmittance	OM	OC × 1.6
EC	Thermal-optical transmittance	EC	Direct
Al	ICP-OES/MS	Al_2O_3 equivalent	Stoichiometric
Ca	ICP-OES/MS	CaO equivalent	Stoichiometric
Fe	ICP-OES/MS	Fe_2O_3 equivalent	Stoichiometric
K	ICP-OES/MS	K_2O equivalent	Stoichiometric
Mg	ICP-OES/MS	MgO equivalent	Stoichiometric
Mn	ICP-OES/MS	MnO equivalent	Stoichiometric
Na	ICP-OES/MS	Na_2O equivalent	Stoichiometric
P	ICP-OES/MS	P_2O_5 equivalent	Stoichiometric
Si	ICP-OES/MS	SiO_2 equivalent	Stoichiometric
Ti	ICP-OES/MS	TiO_2 equivalent	Stoichiometric
As	ICP-OES/MS	As_2O_3 equivalent	Stoichiometric
Ba	ICP-OES/MS	BaO equivalent	Stoichiometric
Be	ICP-OES/MS	BeO equivalent	Stoichiometric
Bi	ICP-OES/MS	Bi_2O_5 equivalent	Stoichiometric
Cd	ICP-OES/MS	CdO equivalent	Stoichiometric
Ce	ICP-OES/MS	CeO_2 equivalent	Stoichiometric
Co	ICP-OES/MS	CoO equivalent	Stoichiometric
Cr	ICP-OES/MS	Cr_2O_3 equivalent	Stoichiometric
Cs	ICP-OES/MS	Cs_2O equivalent	Stoichiometric
Cu	ICP-OES/MS	CuO equivalent	Stoichiometric
Dy	ICP-OES/MS	Dy_2O_3 equivalent	Stoichiometric
Er	ICP-OES/MS	Er_2O_3 equivalent	Stoichiometric
Eu	ICP-OES/MS	Eu_2O_3 equivalent	Stoichiometric
Ga	ICP-OES/MS	Ga_2O_3 equivalent	Stoichiometric
Gd	ICP-OES/MS	Gd_2O_3 equivalent	Stoichiometric
Ge	ICP-OES/MS	GeO_2 equivalent	Stoichiometric
Hf	ICP-OES/MS	HfO_2 equivalent	Stoichiometric

Ho	ICP-OES/MS	Ho_2O_3 equivalent	Stoichiometric
La	ICP-OES/MS	La_2O_3 equivalent	Stoichiometric
Li	ICP-OES/MS	Li_2O equivalent	Stoichiometric
Lu	ICP-OES/MS	Lu_2O_3 equivalent	Stoichiometric
Mo	ICP-OES/MS	MoO_3 equivalent	Stoichiometric
Nb	ICP-OES/MS	Nb_2O_5 equivalent	Stoichiometric
Nd	ICP-OES/MS	Nd_2O_3 equivalent	Stoichiometric
Ni	ICP-OES/MS	NiO equivalent	Stoichiometric
Pb	ICP-OES/MS	PbO equivalent	Stoichiometric
Pr	ICP-OES/MS	Pr_6O_{11} equivalent	Stoichiometric
Rb	ICP-OES/MS	Rb_2O equivalent	Stoichiometric
Sb	ICP-OES/MS	Sb_2O_5 equivalent	Stoichiometric
Sc	ICP-OES/MS	Sc_2O_3 equivalent	Stoichiometric
Se	ICP-OES/MS	SeO_3 equivalent	Stoichiometric
Sm	ICP-OES/MS	Sm_2O_3 equivalent	Stoichiometric
Sn	ICP-OES/MS	SnO_2 equivalent	Stoichiometric
Sr	ICP-OES/MS	SrO equivalent	Stoichiometric
Tb	ICP-OES/MS	Tb_4O_7 equivalent	Stoichiometric
Th	ICP-OES/MS	ThO_2 equivalent	Stoichiometric
Tl	ICP-OES/MS	Tl_2O_3 equivalent	Stoichiometric
Tm	ICP-OES/MS	Tm_2O_3 equivalent	Stoichiometric
U	ICP-OES/MS	UO_2 equivalent	Stoichiometric
V	ICP-OES/MS	V_2O_5 equivalent	Stoichiometric
W	ICP-OES/MS	WO_3 equivalent	Stoichiometric
Y	ICP-OES/MS	Y_2O_3 equivalent	Stoichiometric
Yb	ICP-OES/MS	Yb_2O_3 equivalent	Stoichiometric
Zn	ICP-OES/MS	ZnO equivalent	Stoichiometric
Zr	ICP-OES/MS	ZrO_2 equivalent	Stoichiometric
SO_4^{2-}	IC	WSI	Added
NO_3^-	IC	WSI	Added
NH_4^+	IC	WSI	Added
Cl^-	IC	WSI	Added
F^-	IC	WSI	Added
Br^-	IC	WSI	Added
NO_2^-	IC	WSI	Added
H_2O	Köhler-based calculation	Estimated particle-bound water	Estimated

Supplementary Methods S1. Microtox® data reduction

Bioluminescence inhibition was calculated following the Microtox data-reduction procedure. Luminescence readings were control-corrected using R_t (Eq. 1), converted to gamma response (Γ_t) (Eq. 2), and expressed as percentage effect ($\%E_t$) (Eq. 3). EC_{50} values, expressed as % (v/v) of the extract, were calculated by the Microtox Model 500 analyser from the concentration–gamma regression (Eq. 4), with EC_{50} corresponding to $\Gamma = 1$, i.e., 50% bioluminescence inhibition (Eq. 5).

$$R_t = \frac{I_{t,c}}{I_{0,c}} \quad \text{(Eq. 1)}$$

$$\Gamma_t = \frac{R_t I_0 - I_t}{I_t} \quad \text{(Eq. 2)}$$

$$\%E_t = \frac{\Gamma_t}{1+\Gamma_t} \times 100 \quad \text{(Eq. 3)}$$

$$\log\ C = b \log\ \Gamma + \log\ a \quad \text{(Eq. 4)}$$

$$EC_{50} = C_{\Gamma=1} = 10^{\log a} \quad \text{(Eq. 5)}$$

where I_0 and I_t are the initial and post-exposure luminescence of the sample, respectively; $I_{0,c}$ and $I_{t,c}$ are the corresponding control readings; C is the extract concentration expressed as % (v/v); and a and b are regression parameters.

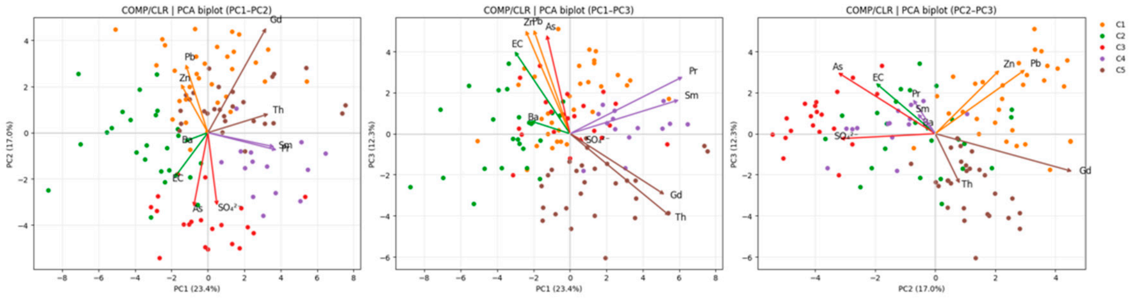

Figure S1. PCA biplots of COMP/CLR data by cluster: score plots coloured by cluster (C1–C5) with loading vectors for selected species (highest absolute loadings in the plotted principal-component (PC) planes). Panels: (a) PC1–PC2, (b) PC1–PC3, (c) PC2–PC3.

Table S3. Quantitative assessment of cluster-number solutions using internal validity indices (k = 2–10)

K	*SILHOUETTE COEFFICIENT*	*CALINSKI–HARABASZ INDEX*	*DAVIES–BOULDIN INDEX*
2	0.129	18.4	2.40
3	0.150	19.2	1.93
4	0.158	20.2	1.77
5	0.162	19.7	1.61
6	0.156	18.2	1.54
7	0.148	17.4	1.67
8	0.156	16.9	1.60
9	0.152	16.2	1.63
10	0.160	15.8	1.57

Table S4. Resampling-based assessment of global stability for four-, five- and six-cluster solutions using 80% of the observations.

K	*MEAN ARI*
4	0.508
5	0.599
6	0.527

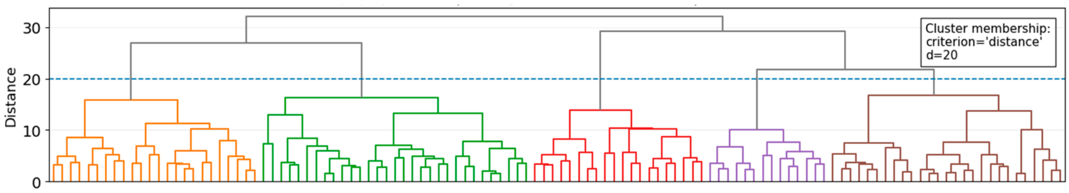

Figure S2. PCA–Ward dendrogram of daily PM_{10} chemical composition. Ward linkage on Euclidean distances in PCA score space; clusters (C1–C5) defined by a fixed distance cut (d = 20; dashed line), with coloured branches indicating cluster membership.

Table S5. Resampling-based cluster-specific stability of the retained five-cluster solution under 80% subsampling. *N* denotes the number of observations assigned to each cluster in the full-data solution.

CLUSTER	*N*	*MEAN JACCARD SIMILARITY*
C1	24	0.59
C2	31	0.50
C3	20	0.87
C4	14	0.68
C5	27	0.79

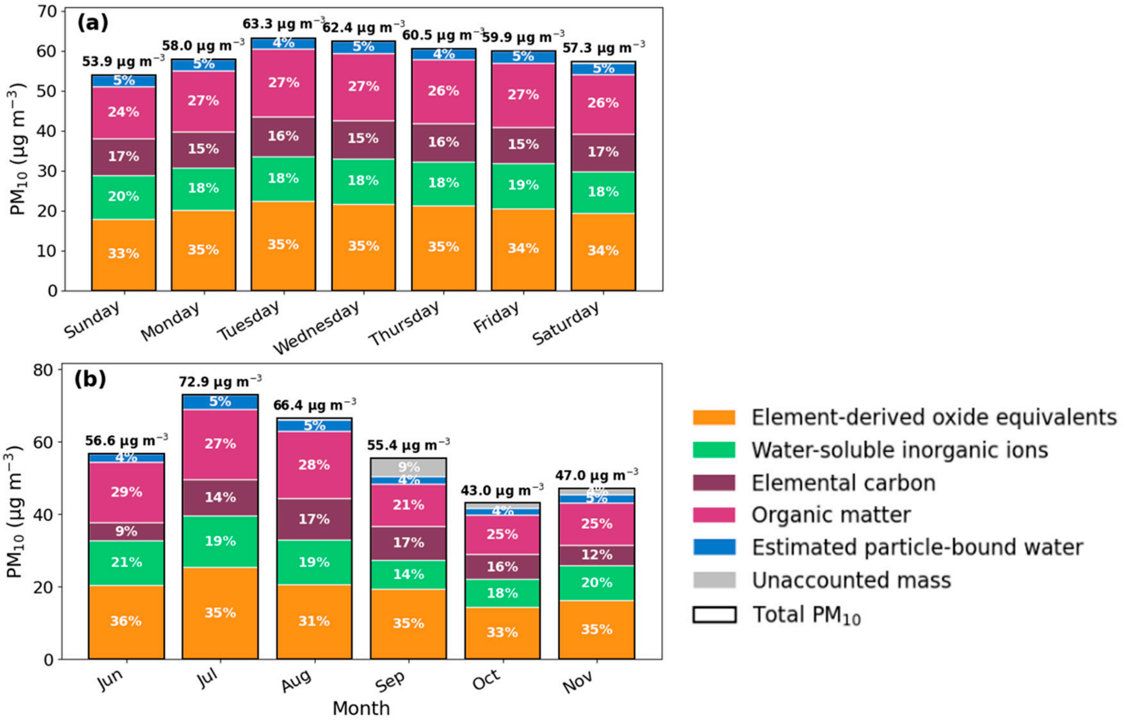

Figure S3. Temporal variation in PM_{10} chemical composition by major classes. Stacked mass percentages and corresponding mean PM_{10} concentrations (µg m^{-3}) stratified by (a) day of week and (b) month.

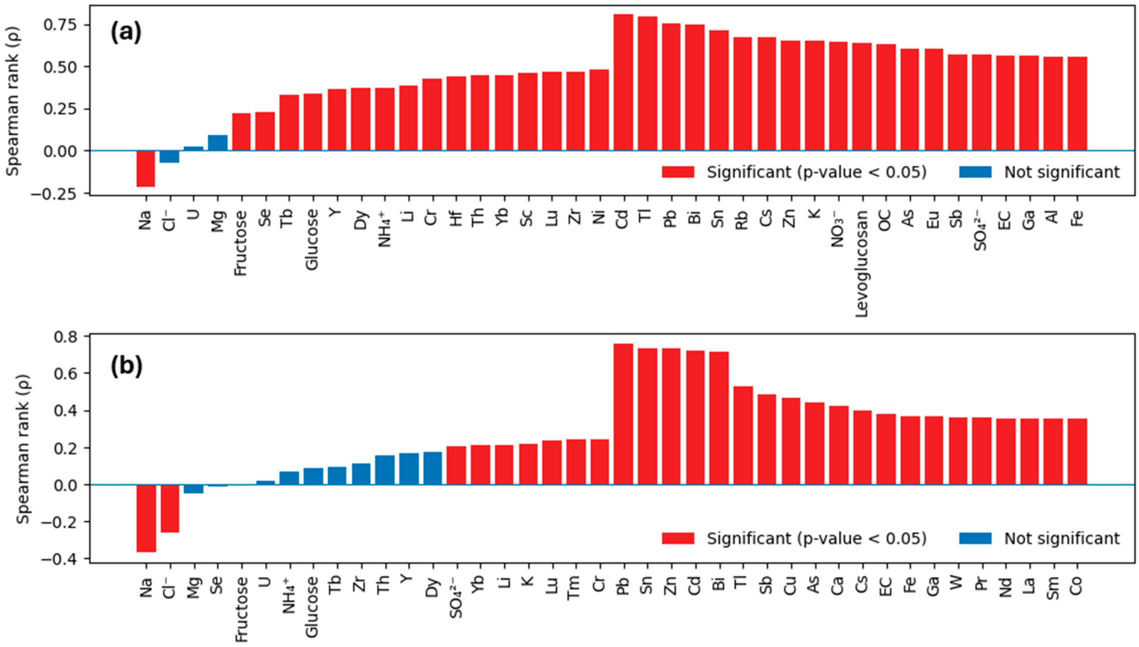

Figure S4. Spearman rank correlations (ρ_S) between each chemical species and TU15 (a) and ΔTU (b) , shown for the top 20 species positive and negative rho. Bars are coloured by multiple-testing significance after Benjamini–Hochberg FDR correction (red: p_fdr < 0.05; blue: not significant)

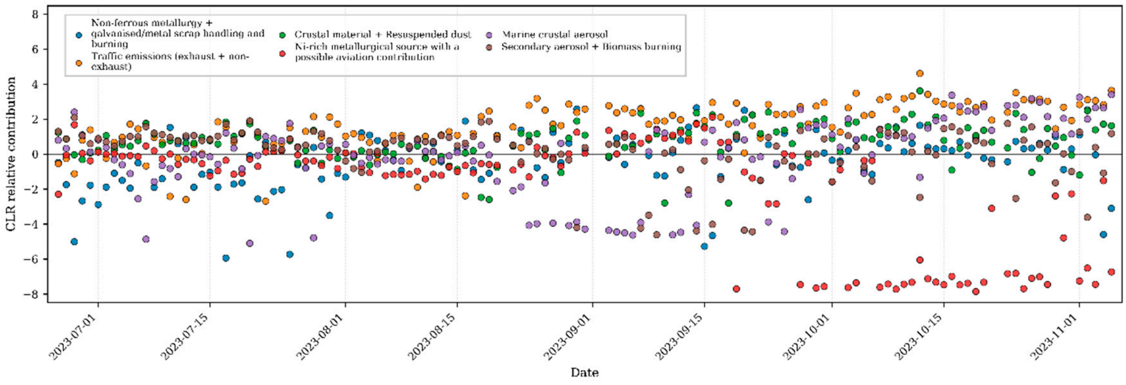

Figure S5. Temporal evolution of CLR-relative contributions of PMF-resolved sources in daily PM_{10} samples from Luanda, Angola.

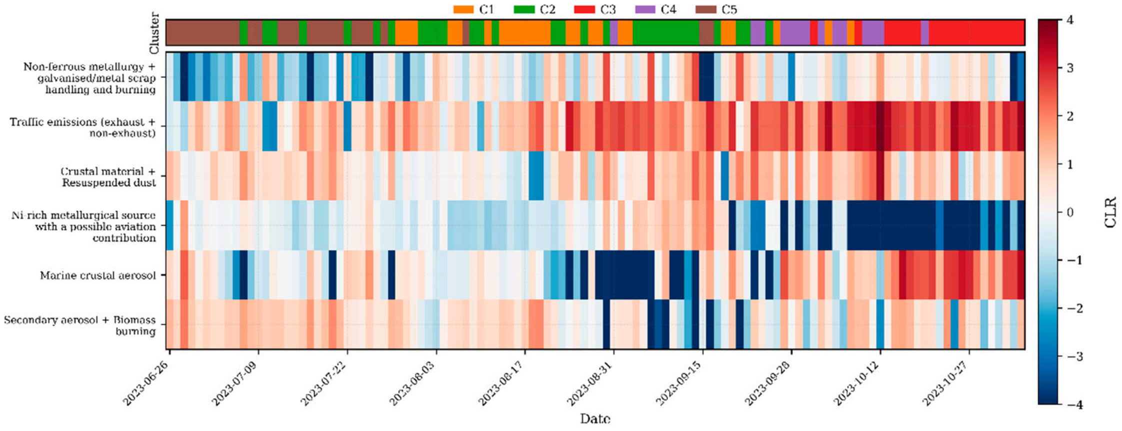

Figure S6. Heatmap of CLR-relative PMF source contributions across daily PM_{10} chemical regimes. Columns represent date-ordered samples from Luanda, Angola, the upper bar indicates PCA–Ward cluster membership (C1–C5), and colours denote relative enrichment or depletion of each PMF-resolved source.
